# Association of germline *ABCB1* and *ERCC1* polymorphisms with CDK4/6 inhibitor-induced neutropenia in patients with breast cancer: a systematic review and meta-analysis

**DOI:** 10.3389/fphar.2026.1872557

**Published:** 2026-08-25

**Authors:** Marina-Daniela Dimulescu, Cristian Virgil Lungulescu, Anca-Lelia Riza, Ioana Streata, Radu Antoniu Patrascu, Anca-Maria Capraru, Mihaela-Simona Naidin, Bogdan Silviu Ungureanu, Ioana Andreea Gheonea, Adina Turcu-Stiolica

**Affiliations:** 1 Doctoral School, University of Medicine and Pharmacy of Craiova, Craiova, Romania; 2 Oncology Department, Faculty of Medicine, University of Medicine and Pharmacy of Craiova, Craiova, Romania; 3 Department of Genetics, Faculty of Medicine, University of Medicine and Pharmacy of Craiova, Craiova, Romania; 4 Department of Pharmaceutical Management and Marketing, Faculty of Pharmacy, University of Medicine and Pharmacy of Craiova, Craiova, Romania; 5 Department of Gastroenterology, Faculty of Medicine, University of Medicine and Pharmacy of Craiova, Craiova, Romania; 6 Department of Radiology, Faculty of Medicine, University of Medicine and Pharmacy of Craiova, Craiova, Romania; 7 Department of Pharmacoeconomics, Faculty of Pharmacy, University of Medicine and Pharmacy of Craiova, Craiova, Romania; 8 Department of Health Economics and Outcomes Research, Faculty of Medicine, University of Medicine and Pharmacy “Iuliu Hatieganu”, Craiova, Romania

**Keywords:** *ABCB1*, cancer, CDK4/6 inhibitor, *ERCC1*, neutropenia, palbociclib, pharmacogenomics

## Abstract

**Background:**

CDK4/6 inhibitors are standard-of-care for HR+/HER2− advanced breast cancer, but grade 3/4 neutropenia occurs in up to 60% of patients. Germline polymorphisms in *ABCB1*, encoding the P-glycoprotein efflux transporter, have been investigated as predictors of CDK4/6 inhibitor–induced neutropenia, with conflicting results across populations. No meta-analysis has previously pooled these findings.

**Methods:**

PubMed, Scopus, and Web of Science were systematically searched. Studies reporting genotype-stratified grade 3/4 neutropenia in CDK4/6 inhibitor–treated patients were eligible. Four SNPs were analyzed: *ABCB1* rs1128503, *ABCB1* rs1045642, *ERCC1* rs11615, and *ERCC1* rs3212986. Odds ratios were pooled using Mantel-Haenszel weighting with random-effects (rs1128503, rs11615) or fixed-effect (rs1045642, rs3212986) models under dominant genetic models, stratified by ancestry. The protocol was registered in PROSPERO (CRD420261379298).

**Results:**

Four studies (1,138 patients) were included. No SNP showed a significant overall association. However, significant ancestry-dependent heterogeneity was detected for two *ABCB1* SNPs. For rs1045642, T-carrier status was significantly associated with increased neutropenia in European/Caucasian patients (pooled OR 1.62, 95% CI 1.05–2.52, p = 0.03, I^2^ = 0%) but decreased risk in East Asians (OR 0.31, 95% CI 0.13–0.74, p = 0.009; subgroup difference p = 0.0009). For rs1128503, a concordant European trend was observed (OR 1.87, 95% CI 0.87–4.00, p = 0.11) with reversed direction in North African/Middle Eastern patients (OR 0.33, 95% CI 0.12–0.92, p = 0.03; subgroup difference p = 0.02). For *ERCC1* rs11615, the inclusion of Wang 2024 nullified the previously borderline European signal (pooled OR 0.99, 95% CI 0.42–2.33, p = 0.98, I^2^ = 75%), with subgroup differences no longer significant (p = 0.17). *ERCC1* rs3212986 showed no association (OR 1.07, 95% CI 0.53–2.17, I^2^ = 0%).

**Conclusion:**

These preliminary findings suggest that *ABCB1* rs1045642 T-carrier status may be associated with CDK4/6 inhibitor–induced neutropenia in European/Caucasian patients, with a concordant trend for rs1128503 findings. Both associations appear ancestry-dependent, although the limited number of studies and single-study ancestry subgroups preclude definitive conclusions. *ERCC1* rs11615, previously borderline in a single study, was not confirmed upon independent replication. Prospective validation in adequately powered, ancestry-stratified cohorts incorporating *ABCB1* haplotype analysis and pharmacokinetic sampling is warranted.

**Systematic Review Registration:**

identifier CRD420261379298.

## 1 Introduction

Breast cancer remains the most commonly diagnosed malignancy among women worldwide, with an estimated 2.3 million new cases in 2022 (Bray et al., 2024). Among the molecular subtypes, hormone receptor-positive (HR+), human epidermal growth factor receptor 2-negative (HER2−) disease accounts for approximately 70% of all breast cancers and represents the largest subgroup of patients with advanced or metastatic disease (Cardoso et al., 2018). Over the past decade, the treatment landscape for HR+/HER2− advanced breast cancer has been transformed by the development of cyclin-dependent kinase four and six inhibitors (CDK4/6i) — palbociclib, ribociclib, and abemaciclib—which, in combination with endocrine therapy, have demonstrated significant improvements in progression-free survival and, for ribociclib and abemaciclib, overall survival (Finn et al., 2016; Hortobagyi et al., 2016; Goetz et al., 2017; Sledge et al., 2020). These agents are now established as the standard of care in first- and second-line settings, as endorsed by international guidelines (Rugo et al., 2016; Gennari et al., 2021).

Despite their clinical benefit, CDK4/6i are associated with a high incidence of hematologic toxicity, with neutropenia representing the most frequent treatment-related adverse event. In the pivotal registration trials, grade 3 or 4 neutropenia occurred in approximately 55%–67% of patients treated with palbociclib or ribociclib, and in 25%–36% of patients receiving abemaciclib (Finn et al., 2016; Hortobagyi et al., 2016; Goetz et al., 2017; Sledge et al., 2020; Diéras et al., 2019a). Although CDK4/6i–induced neutropenia is mechanistically distinct from chemotherapy-induced myelosuppression - involving reversible G1 cell cycle arrest of hematopoietic progenitor cells rather than apoptotic cell death (Hu et al., 2016) - it remains the primary cause of dose interruptions, dose reductions, and treatment discontinuations, affecting 35%–45% of patients (Diéras et al., 2019a; Ettl et al., 2020). Such treatment modifications may compromise dose intensity and, potentially, long-term treatment adherence and outcomes (Roncato et al., 2023; Lungulescu et al., 2024; Turcu-Stiolica et al., 2024).

A notable observation from the CDK4/6i clinical program is the substantially higher prevalence and severity of neutropenia among Asian patients. Subgroup analyses from the PALOMA trials demonstrated that grade 4 neutropenia occurred in 18%–34% of Asian patients compared with approximately 8% of non-Asian patients (Peruzzi et al., 2023; Mukai et al., 2019; Iwata et al., 2021). Similarly, the PALOMA-4 trial, conducted exclusively in Asian postmenopausal women, reported any-grade neutropenia in 98.2% of patients (Xu et al., 2022). Several clinical risk factors have been identified to explain this interindividual and interethnic variability, most notably low baseline absolute neutrophil count (ANC), which has consistently emerged as the strongest predictor of severe neutropenia across multiple studies and populations (Peruzzi et al., 2023; Iwata et al., 2021; Diéras et al., 2019b; Verma et al., 2016; Ibrahim Mohamed Ebid et al., 2025). Lower body mass index and younger age have also been implicated, though less consistently (Peruzzi et al., 2023; Iwata et al., 2021).

Beyond clinical risk factors, germline genetic variation in genes governing drug absorption, distribution, metabolism, and elimination (ADME) has attracted interest as a potential contributor to the interindividual variability in CDK4/6i toxicity (Roncato et al., 2020). All three CDK4/6i are metabolized primarily by cytochrome P450 3A (CYP3A) enzymes and are substrates of the efflux transporter P-glycoprotein (P-gp), encoded by the adenosine triphosphate-binding cassette subfamily B member 1 gene (*ABCB1*) (de Gooijer et al., 2015; Martínez-Chávez et al., 2019; Martínez-Chávez et al., 2022; Sorf et al., 2018). P-gp is expressed at the apical membrane of intestinal enterocytes, hepatocytes, and renal tubular cells, where it limits the oral bioavailability of substrate drugs by extruding them back into the intestinal lumen and facilitating hepatobiliary excretion (Hodges et al., 2011). Common single nucleotide polymorphisms (SNPs) in *ABCB1* - particularly rs1128503 (c.1236C>T), rs1045642 (c.3435C>T), and rs2032582 (c.2677G>T/A) - have been associated with altered P-gp expression and function, especially when co-inherited as the 1236T–2677T(A)–3435T haplotype (Hoffmeyer et al., 2000; Salama et al., 2006; Vivona et al., 2014). These variants have been extensively studied in the context of other P-gp substrate drugs, including imatinib (Harivenkatesh et al., 2017), digoxin (Horinouchi et al., 2002), and cyclosporine (Chowbay et al., 2003), with variable and often population-dependent results.

A separate pharmacogenomic hypothesis involves the excision repair cross-complementing group 1 gene (*ERCC1*), which encodes a key component of the nucleotide excision repair pathway (Friboulet et al., 2013). Genetic variants of *ERCC1* — particularly rs11615 and rs3212986 — have been associated with severe neutropenia in Asian breast cancer patients treated with anthracycline-based chemotherapy, presumably through differential DNA repair capacity affecting hematopoietic progenitor cell susceptibility to cytotoxic damage (Tsuji et al., 2016). Whether these variants similarly influence the susceptibility to CDK4/6i–induced neutropenia - which involves cell cycle arrest rather than DNA damage - is an open question.

To our knowledge, this is the first systematic review and meta-analysis to investigate the association between germline pharmacogenomic polymorphisms and CDK4/6i-related toxicity. The objective of this systematic review and meta-analysis was therefore to identify, critically appraise, and quantitatively synthesize the available evidence on the association between germline SNPs in pharmacokinetically relevant genes, primarily *ABCB1* and *ERCC1*, and CDK4/6i-induced grade 3/4 neutropenia, with pre-specified stratification by population ancestry to address the known inter-ethnic variability.

## 2 Methods

### 2.1 Literature search and study selection

This systematic review and meta-analysis were conducted in accordance with the Preferred Reporting Items for Systematic Reviews and Meta-Analyses (PRISMA) 2020 guidelines (Page et al., 2021) and the Human Genome Epidemiology Network (HuGENet) (Khoury, 1997; Tsuji et al., 2016) recommendations for reporting genetic association meta-analyses (PRISMA checklist is included in Supplementary Table S1). The protocol was prospectively registered in the International Prospective Register of Systematic Reviews (PROSPERO; registration number CRD420261379298).

The PICOS (PECOS framework) is clearly defined as:

Population (P) = Adult patients (≥18 years) with cancer, predominantly HR+/HER2− advanced or metastatic breast cancer, who received at least one dose of a CDK4/6 inhibitor (palbociclib, ribociclib, or abemaciclib) in combination with endocrine therapy.

Intervention (exposure, I/E) = Germline single-nucleotide polymorphism genotype in ≥1 pharmacokinetically relevant gene—specifically, carriage of the variant allele (dominant model) in the prespecified SNPs: *ABCB1* rs1128503, rs1045642, rs2032582; *ERCC1* rs11615, rs3212986; *ABCG2* rs2231142; CYP3A4*22; CYP3A5*3; *SULT2A1* rs182420; and the *ABCB1* 1,236T–2677T(A)–3435T haplotype.

Comparator (C) = The corresponding wild-type/reference genotype for each SNP (common homozygotes), harmonized across studies according to the risk-allele direction reported in each study.

Outcome (O) = Primary: grade 3/4 neutropenia (CTCAE v4.0/5.0), preferably assessed at ∼ day 14–15 of cycle 1. Secondary: cumulative grade 3/4 neutropenia at any time during treatment; dose-limiting toxicities within cycle 1; dose reductions within the first three cycles; and treatment withdrawal/discontinuation due to toxicity.

Study designs (S) = Included: randomized controlled trials (including *post hoc* pharmacogenetic analyses), prospective cohort studies, and retrospective observational studies reporting genotype-stratified toxicity, with data sufficient to construct a 2 × 2 table or reporting an OR/RR/HR with 95% CI. Excluded: preclinical/*in vitro* studies; population allele-frequency studies without patient-level genotype–toxicity data; case reports/series with <10 genotyped patients; somatic (tumour) mutation studies; and reviews, editorials, conference abstracts without extractable data, and clinical guidelines.

A systematic literature search was conducted in PubMed/MEDLINE, Scopus, Cochrane Central Register of Controlled Trials (CENTRAL), and Web of Science Core Collection from inception through 30 April 2026. No language or date restrictions were applied. The search strategy combined three concept blocks using Boolean operators: (1) Drug (“palbociclib” OR “ribociclib” OR “abemaciclib”); (2) Pharmacogenomics (pharmacogenet* OR pharmacogenomic* OR polymorphism* OR SNP OR “single nucleotide” OR genotype OR allele OR haplotype OR *ABCB1* OR *ERCC1* OR *ABCG2* OR *CYP3A4* OR *CYP3A5* OR *SULT2A1* OR ″P-glycoprotein” OR *MDR1*); (3) Outcome (neutropenia OR toxicity OR “adverse event” OR “adverse effect” OR “dose-limiting” OR “dose reduction” OR hematologic OR hematotoxicity OR tolerability). Blocks were combined as Block 1 AND Block 2 AND Block 3. Reference lists of included studies and relevant review articles were manually screened.

The population was the adult patients (≥18 years) with cancer - predominantly hormone receptor-positive (HR+), human epidermal growth factor receptor 2-negative (HER2−) advanced or metastatic breast cancer - treated with at least one dose of a CDK4/6i in combination with endocrine therapy.

The exposure was the germline single nucleotide polymorphism (SNP) genotype in at least one pharmacokinetically relevant gene. The primary SNPs of interest were *ABCB1* rs1128503 (c.1236C>T), *ABCB1* rs1045642 (c.3435C>T), *ABCB1* rs2032582 (c.2677G>T/A), *ERCC1* rs11615, *ERCC1* rs3212986, *ABCG2* rs2231142 (c.421C>A), CYP3A4*22 (rs35599367), CYP3A5*3 (rs776746), *SULT2A1* rs182420, and the *ABCB1* three-SNP haplotype (1,236T–3435T–2677T [A]). Additional SNPs reported in eligible studies were also considered. These genes were selected *a priori* based on the known absorption, distribution, metabolism, and elimination (ADME) pathways of CDK4/6 inhibitors: *ABCB1* encodes P-glycoprotein, a key efflux transporter for which all three CDK4/6 inhibitors are established substrates; *ERCC1* encodes a nucleotide excision repair protein whose variants have been associated with chemotherapy-induced neutropenia; and *ABCG2*, *CYP3A4*, *CYP3A5*, and *SULT2A1* encode additional drug-metabolizing enzymes or transporters involved in CDK4/6 inhibitor pharmacokinetics. Quantitative pooling was ultimately feasible only for the four SNPs (*ABCB1* rs1128503, *ABCB1* rs1045642, *ERCC1* rs11615, *ERCC1* rs3212986) for which two or more independent study populations reported extractable genotype-stratified neutropenia data. The search strategy encompassed all three approved CDK4/6 inhibitors (palbociclib, ribociclib, and abemaciclib); however, no published study reporting genotype-stratified grade 3/4 neutropenia data for abemaciclib-treated patients met our inclusion criteria at the time of search, likely reflecting abemaciclib’s lower incidence of severe neutropenia and the earlier stage of pharmacogenomic research for this agent.

The comparator was the wild-type or reference genotype for each SNP. The specific reference allele was harmonized across studies based on the risk allele direction reported in each study.

The primary outcome was grade 3/4 neutropenia, defined per the Common Terminology Criteria for Adverse Events (CTCAE) version 4.0 or 5.0, preferably assessed at approximately day 14–15 of the first treatment cycle. Secondary outcomes included cumulative grade 3/4 neutropenia at any time during treatment, dose-limiting toxicities (DLTs) within the first cycle, dose reductions within the first three cycles, and drug withdrawal or discontinuation due to toxicity. Studies must report sufficient data to construct a 2 × 2 table (events and totals by genotype group) or report an odds ratio, risk ratio, or hazard ratio with 95% confidence interval for the genotype–toxicity association.

Randomized controlled trials (including *post hoc* pharmacogenetic analyses), prospective cohort studies, and retrospective observational studies reporting genotype-stratified toxicity outcomes were eligible. Studies were required to report sufficient data to construct a 2 × 2 table (events and totals by genotype group) or to report an odds ratio (OR), risk ratio (RR), or hazard ratio (HR) with 95% confidence interval (CI) for the genotype–toxicity association.

Studies were excluded if they were preclinical or *in vitro* investigations; population-level allele frequency studies without patient-level genotype–toxicity data; case reports or case series with fewer than 10 genotyped patients; studies examining somatic (tumor) mutations only; reviews, editorials, conference abstracts without extractable data, or clinical practice guidelines.

Two reviewers (M-D.D. and C.V.L.) independently screened titles and abstracts against the eligibility criteria, followed by full-text assessment of potentially eligible records. Disagreements at either stage were resolved by consensus or by consultation with a third reviewer (A.T-S.). The study selection process was documented using a PRISMA flow diagram. All retrieved records were imported into Rayyan, a systematic review software platform, to facilitate screening and reference management. Duplicate entries were removed using electronic de-duplication functions, and the absence of remaining duplicates was subsequently confirmed through manual verification.

### 2.2 Data extraction and quality assessment

The data extraction was employed to capture the following information from each eligible study. Study-level data included first author, year of publication, country, study design, clinical trial name (where applicable), sample size, population ancestry or self-reported ethnicity, specific CDK4/6i used, endocrine therapy partner, treatment line, and version of the Common Terminology Criteria for Adverse Events (CTCAE) utilized for grading adverse events. Genotype-level data comprised gene name, SNP rs number, genotype frequencies (homozygous wild-type, heterozygous, homozygous variant), allele frequencies, Hardy-Weinberg equilibrium (HWE) status and corresponding p-value, genotyping platform, and quality control parameters (call rate, concordance with re-genotyping or sequencing). Outcome data included the number of events (grade 3/4 neutropenia) and total patients per genotype group, the applied genetic model (dominant, recessive, codominant, or additive), odds ratios or risk ratios with 95% confidence intervals (CIs), specification of whether estimates were unadjusted (univariate) or adjusted (multivariable), and the covariates incorporated into multivariable models. For studies reporting per-genotype event counts without corresponding group totals (i.e., providing only the genotype distribution among cases), the total number of patients per genotype group was back-calculated from the reported univariate odds ratio and the known marginal totals (total eligible patients and total events), leveraging the algebraic relationship between the odds ratio, event counts, and non-event counts. For studies reporting exclusively three-genotype (codominant) data, groups were collapsed to conform to the dominant model (variant-allele carriers versus homozygous wild-type) employed in the primary analysis. This was achieved by summing events and totals for the heterozygous and homozygous variant groups.

The genotype table reconstruction procedure was as follows. For Iwata 2021, which reported per-genotype event counts without corresponding group totals, the non-event count for each genotype group was derived algebraically by solving OR = (a/b)/(c/d), where a and c are the known event counts and b and d are the unknown non-event counts, subject to the constraint that total patients equal the sum of events and non-events across genotype groups. For Wang 2024, which reported codominant odds ratios and genotype group totals but not event counts, the 2 × 2 table was reconstructed by solving the system of equations relating the odds ratio to events and non-events for each genotype group, followed by collapsing heterozygous and homozygous variant groups to match the dominant model. These reconstructions assume that the reported odds ratios are exact (not rounded beyond two decimal places), that marginal totals are accurately reported, and that no continuity corrections were applied in the original analyses. Where both reconstructed and directly reported data were available (e.g., Iwata’s Asian subgroup for *ABCB1* SNPs), the reconstructed tables were cross-checked against the reported event rates and found to be internally consistent. Minor rounding differences in reported ORs could introduce small discrepancies in reconstructed cell counts, but these would not materially alter the pooled estimates.

Risk of bias was independently assessed by two reviewers using two complementary instruments (M-D.D. and A-L.R.). The Q-GENIE (Quality of Genetic Association Studies) was selected as the primary quality assessment tool because it is specifically designed for genetic association studies and evaluates domains unique to this study type - including Hardy-Weinberg equilibrium testing, appropriateness of genetic model assumptions, and control for population stratification - that are not captured by general-purpose tools such as the Newcastle–Ottawa Scale (NOS). Because all included studies were candidate-gene association studies, a genetics-specific instrument was considered essential for a valid appraisal of methodological quality, and Q-GENIE was therefore designated the primary tool while the NOS served only as a general epidemiological cross-check. The NOS was used as a complementary instrument to provide a supplementary assessment of study design quality from a broader epidemiological perspective. The Q-GENIE instrumentevaluated 11 domains on a 1–7 Likert scale: study rationale; selection and definition of the outcome; selection and comparability of comparison groups; technical and non-technical classification of exposure; other sources of bias; sample size and power; *a priori* planning of analyses; statistical methods and control for confounding; testing of assumptions for genetic analyses (including Hardy-Weinberg equilibrium and linkage disequilibrium); and appropriateness of inferences. Total scores were used to classify studies as having low risk of bias (>45), moderate risk (36–45), or high risk (≤35). The Newcastle-Ottawa Scale (NOS) for cohort studies was used as a supplementary assessment of study design quality, scoring selection (4 stars), comparability (2 stars), and outcome ascertainment (3 stars), for a maximum total of nine stars (low risk ≥7, moderate 5–6, high ≤4). Disagreements between reviewers were resolved by consensus.

### 2.3 Statistical analysis

The primary effect measure was the odds ratio (OR) computed from 2 × 2 genotype × event tables using Mantel-Haenszel weighting. For the *ABCB1* rs1128503 analysis, which included studies with opposing effect directions across populations, a random-effects model (DerSimonian-Laird) was used. For the *ERCC1* analyses, which were based on a single study split by ancestry, a fixed-effect model (Mantel-Haenszel) was used.

The primary analysis used a dominant model for the variant allele. For *ABCB1* rs1128503 (c.1236C>T), the comparison was T-allele carriers (CT + TT) versus CC homozygotes. For *ERCC1* rs11615, the comparison was G-allele carriers (GG + AG) versus AA homozygotes. For *ERCC1* rs3212986, the comparison was C-allele carriers (CC + AC) versus AA homozygotes. Where individual studies reported a different genetic model (e.g., recessive), their raw genotype × event data were re-extracted and re-collapsed to match the dominant model used in the pooled analysis.

The dominant model was selected for the primary analysis because it maximizes statistical power when sample sizes are limited, by collapsing heterozygous and homozygous variant carriers into a single group and thereby increasing the number of individuals in each comparison arm. This approach is standard in candidate-gene meta-analyses with few studies and small sample sizes. For all SNPs, the common homozygous genotype served as the reference group (e.g., CC for rs1045642, CC for rs1128503). A limitation of this approach is that the “common” genotype may differ across populations: for example, the rs1128503 CC genotype is less frequent in East Asian than in European populations, which affects the statistical properties of the comparison and contributes to observed heterogeneity across ancestry subgroups. This population-dependent shift in reference group size is discussed further in the Discussion. Additionally, the dominant model may mask recessive effects, which cannot be reliably assessed given the small number of rare homozygotes in several subgroups.

Pre-specified subgroup analyses were conducted by population ancestry, stratified as East Asian, European/Caucasian, and North African/Middle Eastern, based on the self-reported ethnicity of each study population. This stratification was motivated by the substantial inter-ethnic differences in *ABCB1* and *ERCC1* allele frequencies documented in population databases and by evidence from the included studies that the genotype–neutropenia association was confounded by race.

Statistical heterogeneity was assessed using the Cochran Q test (significance threshold p < 0.10) and the I^2^ statistic, interpreted as low (<25%), moderate (25%–75%), or high (>75%). The test for subgroup differences (Chi^2^ test with associated I^2^) was used to evaluate whether the effect differed across ancestry subgroups.

Formal assessment of publication bias (funnel plots, Egger’s test) was not performed because the number of studies per SNP–outcome comparison was fewer than 10 - the minimum recommended for reliable assessment. Publication bias is instead discussed narratively.

All statistical analyses were performed using Review Manager (RevMan) version 5.4 (The Cochrane Collaboration). Forest plots were generated for each poolable SNP–outcome–ancestry combination.

## 3 Results

### 3.1 Search results

**FIGURE 1:**
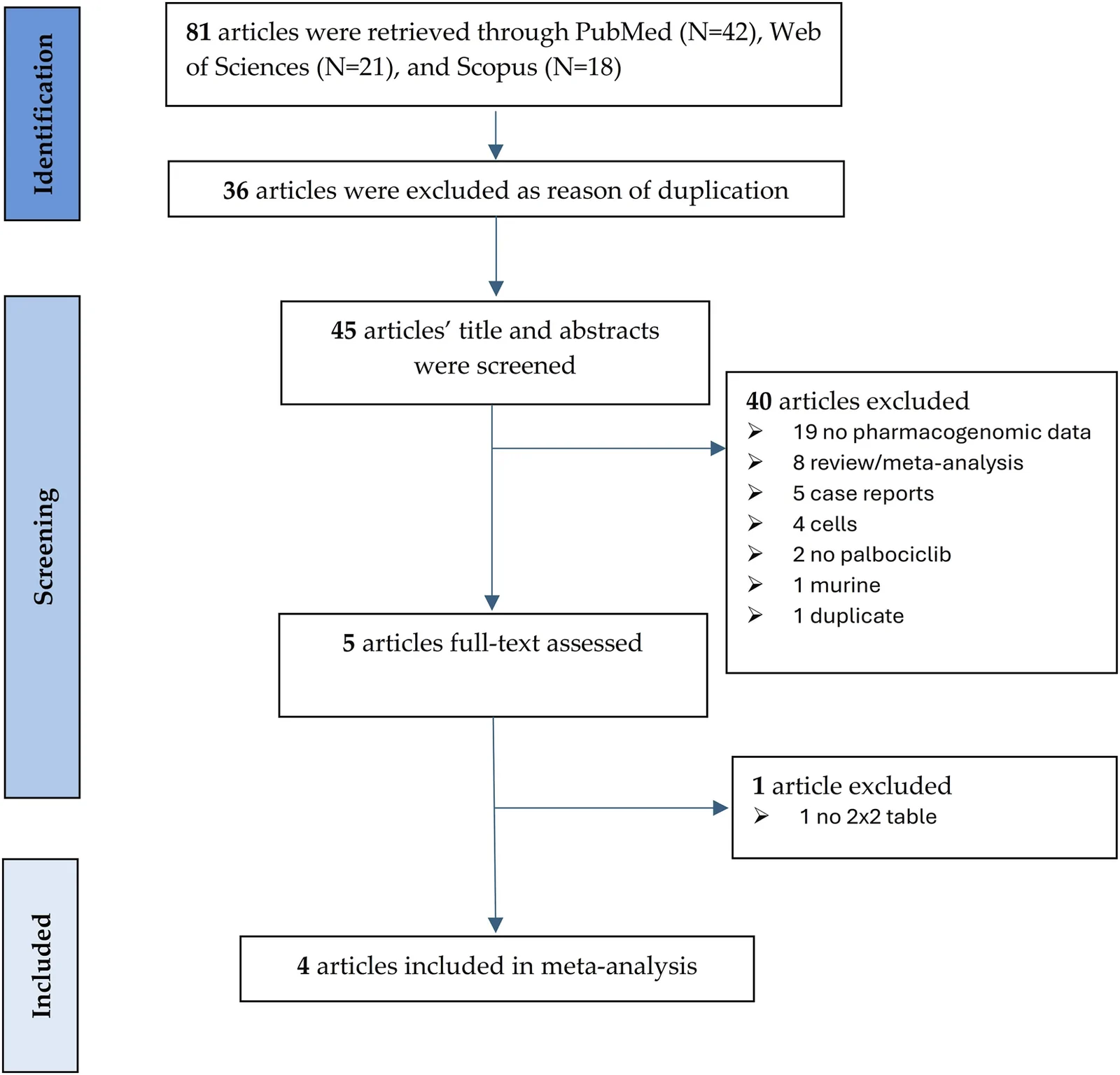
PRISMA 2020 flow diagram of the study selection process for the systematic review and meta-analysis of germline pharmacogenomic polymorphisms and CDK4/6 inhibitor–induced neutropenia.

A total of 81 records were identified through database searching: 42 from PubMed, 21 from Web of Science, and 18 from Scopus. After removal of 36 duplicate records, 45 articles remained for title and abstract screening. Of these, 41 records were excluded for the following reasons: absence of pharmacogenomic data (n = 19), review or meta-analysis design (n = 8), case reports (n = 5), cell-based studies (n = 4), no palbociclib exposure (n = 2), murine study (n = 1), and duplicate record (n = 1). Five full-text articles were assessed for eligibility. One article was excluded because of the absence of extractable 2 × 2 table data. Therefore, four studies were included in the final meta-analysis, as shown in Figure 1 (15, 17, 21, 38) and Table 1. The restriction to palbociclib and ribociclib was not by design but by data availability. Our systematic search was designed to capture all three approved CDK4/6 inhibitors (palbociclib, ribociclib, and abemaciclib). However, at the time of our search (through April 2026), no published study reported genotype-stratified grade 3/4 neutropenia data for abemaciclib-treated patients that met our inclusion criteria.

**TABLE 1:** Main characteristics of studies included in the systematic review and meta-analysis of germline pharmacogenomic polymorphisms and CDK4/6 inhibitor-related toxicity.

Study	Country Sample size	Ancestry	Study design	CDK 4/6 inhibitor Endocrine therapy partner	Genotyping method HWE status	Toxicity grading	Timing of assessment	Adjustment variables	Risk of bias Q-GENIE tool (max 77) NOS (max 9)
Peruzzi et al. (2023)	Italy (n = 195)	European/Caucasian	Prospective single-centre cohort study	Palbo (59%); Ribo (33%); Abema (8%) Letrozole: (61.3%); fulvestrant: (36.9%); exemestane (0.9%); anastrozole: (0.9%)	Kompetitive allele–specific assays (LGC Genomics, Hoddesdon, United Kingdom); pyrosequencing was used for *ABCB1* c.2677G>T/A to allow triallelic discrimination No significant deviation from HWE was reported for the analysed SNPs	CTCAE v5.0	day 14 of the first treatment cycle	For neutropenia and early DLTs: index CDK4/6 inhibitor and baseline ANC; for dose reduction: index CDK4/6 inhibitor and age	60 (LR) 9 (LR)
Iwata et al. (2021)	Asia (n = 122) non-Asia (n = 530)	East Asian and non-Asian; the non-Asian group was predominantly self-reported White patients	*post hoc* analysis of phase III RCTs	Palbo Letrozole in PALOMA-2; fulvestrant in PALOMA-3	TaqMan assays on a QuantStudio 12 K Flex Real-Time PCR system All four analysed SNPs were in HWE in both Asian and non-Asian groups	CTCAE v4.0 grading thresholds for grade 3 (ANC 500–1,000/mm^3^) and grade 4 (ANC<500/mm^3^)	Cycle 1 day 15, defined as day 15 ± 1 day of treatment	Univariate and multivariable logistic regression; analyses stratified by Asian versus non-Asian ethnicity	62 (LR) 9 (LR)
Ibrahim Mohamed Ebid et al. (2025)	Egypt (n = 95)	North African/Middle Eastern	Prospective observational cohort study	Palbo Fulvestrant: 64/95 (67.4%); AI: 31/95 (32.6%)	Real-time PCR using a TaqMan SNP genotyping assay Deviated from HWE; *p* = 0.0007	CTCAE v4.0 grading thresholds for grade 3 (ANC 500–1,000/mm^3^) and grade 4 (ANC<500/mm^3^)	Cumulative during Palbo treatment; not restricted to day 14/15	Genotype–toxicity association was assessed by univariate logistic regression; no multivariable adjustment for baseline ANC was reported	43 (MR) 7 (MR)
Wang et al. (2024)	United States of America (n = 197	White (90.5%), Black (4.5%), Asian (3.5%), Other (1.0%), Not reported (0.5%) by original study	retrospective pharmacogenetic association study	Palbo	Not specified HWE deviation	CTCAE v5.0	Any time during treatment	The codominant ORs are unadjusted (univariate), derived from logistic regression without covariate adjustment. There is no mention of multivariable adjustment for baseline ANC, age, body weight, or other confounders for the *ERCC1* rs11615 analysis	41 (MR) 7 (MR)

AI, aromatase inhibitor; Palbo, palbociclib; HWE, Hardy–Weinberg equilibrium; CTCAE, common terminology criteria for adverse events; NOS, Newcastle-Ottawa Scale; LR, low risk; MR, moderate risk.

Risk of bias was assessed using Q-GENIE and NOS. Ibrahim Mohamed Ebid lost 2 NOS stars on comparability because the genotype–neutropenia association was analyzed only with univariate logistic regression - there was no adjustment for baseline absolute neutrophil count (ANC), which is the strongest known confounder, or any other covariate. Both Iwata and Peruzzi performed multivariate analyses adjusting for ANC and drug type. The critical quality differentiator across both tools is Q-GENIE Domain 10 (testing of genetic analysis assumptions), where Ibrahim Mohamed Ebid scored 2/7 due to significant Hardy-Weinberg equilibrium (HWE) deviation (p = 0.0007). This is the single most damaging quality issue because HWE departure can indicate genotyping error, sample contamination, or unrecognized population admixture—any of which would invalidate the genotype–outcome association. The authors’ explanation (small sample, selected population) is plausible but cannot be verified without raw genotyping data.

### 3.2 Association between *ABCB1*_rs1045642 and grade 3/4 neutropenia

Two studies (three population entries) were included in the meta-analysis on the association between *ABCB1* rs1045642 and grade 3/4 neutropenia: Iwata 2021, contributing an East Asian (n = 114) and a European/Caucasian (n = 530) subgroup, and Peruzzi 2023 (n = 195, Caucasian, mixed CDK4/6i). Data was analyzed using a dominant model for the T allele (T-carrier [CT + TT] versus CC homozygotes) with Mantel-Haenszel fixed-effect weighting.

**FIGURE 2:**
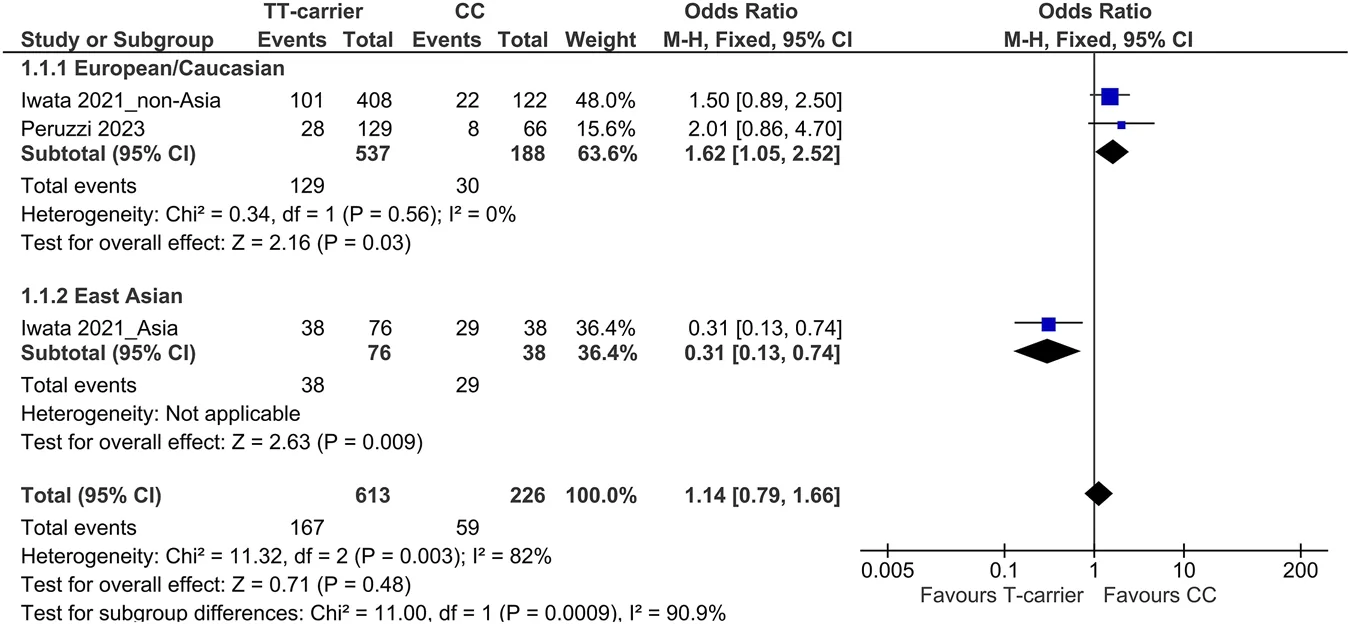
Forest plot of the association between *ABCB1* rs1045642 T-carrier status (CT + TT vs. CC) and grade 3/4 neutropenia in CDK4/6 inhibitor–treated breast cancer patients, stratified by ancestry subgroup (Mantel–Haenszel fixed-effect model).

The overall pooled OR across all three entries (613 T-carriers, 226 CC homozygotes; 226 total events) was 1.14 (95% CI 0.79–1.66, p = 0.48), with high heterogeneity (I^2^ = 82%, Chi^2^ = 11.32, p = 0.003) (Figure 2). The test for subgroup differences was highly significant (Chi^2^ = 11.00, df = 1, p = 0.0009, I^2^ = 90.9%), indicating a strong divergence in the direction of the genotype–neutropenia association between ancestry subgroups.

Two studies contributed to the European subgroup: Iwata non-Asian (101 events among 408 T-carriers versus 22 events among 122 CC; OR 1.50, 95% CI 0.89–2.50) and Peruzzi (28/129 versus 8/66; OR 2.01, 95% CI 0.86–4.70). The pooled subgroup OR was 1.62 (95% CI 1.05–2.52, p = 0.03) with no heterogeneity (I^2^ = 0%, Chi^2^ = 0.34, p = 0.56). This was the only SNP–outcome combination in the entire meta-analysis to reach statistical significance in a pooled subgroup estimate. Both individual study estimates were directionally concordant, with T-carrier status consistently associated with higher neutropenia risk. The neutropenia rate was 24.8% (101/408) in T-carriers versus 18.0% (22/122) in CC homozygotes in Iwata, and 21.7% (28/129) versus 12.1% (8/66) in Peruzzi.

Only Iwata Asian contributed to the Asian subgroup (38/76 T-carriers versus 29/38 CC; OR 0.31, 95% CI 0.13–0.74, p = 0.009). The direction was significantly reversed: CC homozygotes had a much higher neutropenia rate (76.3%) than T-carriers (50.0%). This reversal was statistically significant and drove the overall heterogeneity between subgroups.

### 3.3 Association between *ABCB1*_rs1128503 (c.1236C>T) and grade 3/4 neutropenia

Three studies, comprising a total of 942 patients treated with palbociclib, reported on the association between the *ABCB1* rs1128503 genotype and grade 3/4 neutropenia: Iwata et al. (2021), a pharmacogenetic analysis of the PALOMA-2 and PALOMA-3 trials (n = 652, palbociclib only); Peruzzi et al. (2023), a prospective cohort study conducted at the CRO Aviano, Italy (n = 195 eligible for the neutropenia endpoint, mixed CDK4/6i) (15); and Ibrahim Mohamed Ebid et al. (2025), a prospective cohort study from Cairo, Egypt (n = 95, palbociclib only) (21). The Iwata cohort included both Asian (n = 122) and non-Asian (n = 530) patients and was stratified accordingly (17). Data were pooled using a dominant genetic model (T-allele carriers [CT + TT] versus CC homozygotes), with the T allele treated as the variant allele. Odds ratios were computed from 2 × 2 tables using Mantel-Haenszel weighting with a random-effects model. Analyses were stratified by ancestry into European/Caucasian, East Asian, and North African/Middle Eastern subgroups.

**FIGURE 3:**
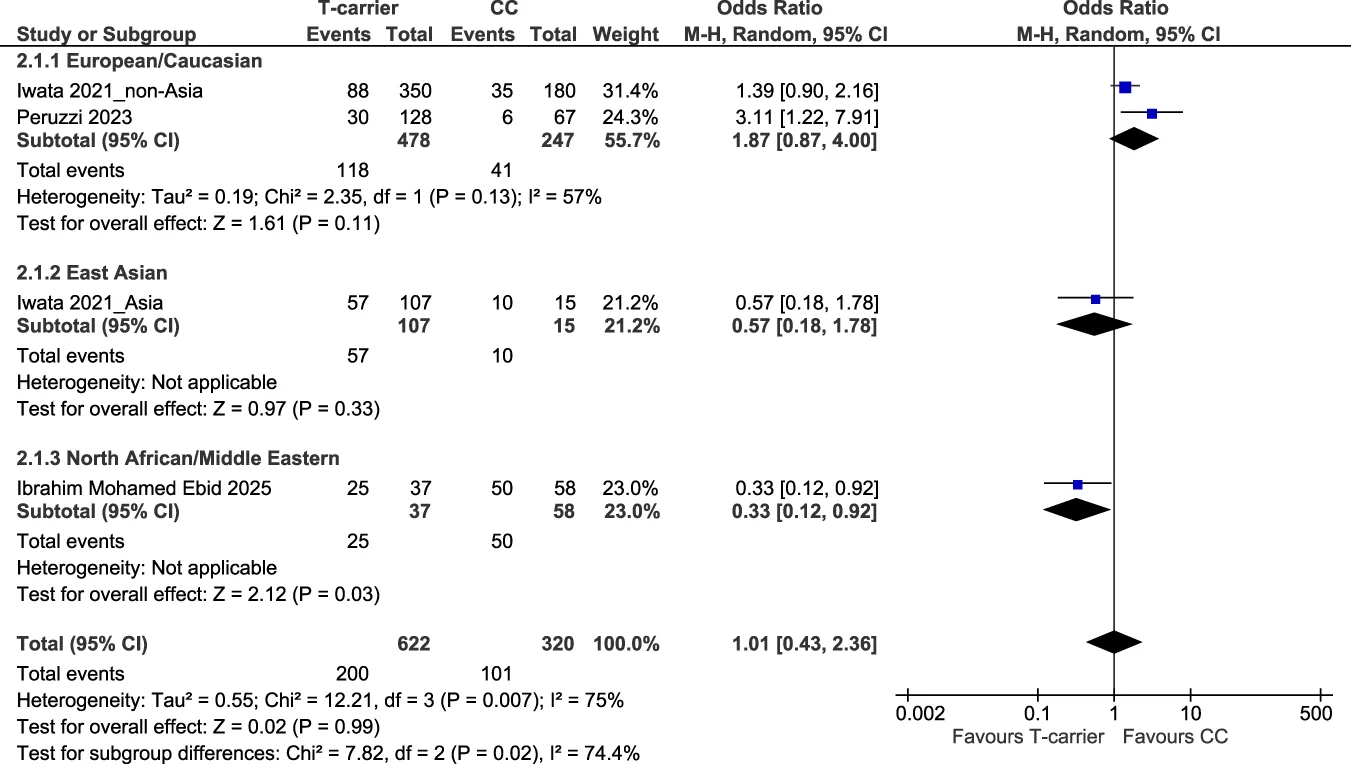
Forest plot of the association between *ABCB1* rs1128503 T-carrier status (CT + TT vs. CC) and grade 3/4 neutropenia in CDK4/6 inhibitor–treated breast cancer patients, stratified by ancestry subgroup (DerSimonian–Laird random-effects model).

The overall pooled OR across all four study–population entries (622 T-carriers, 320 CC homozygotes; 301 total events) was 1.01 (95% CI 0.43–2.36, p = 0.99), indicating no overall association between rs1128503 T-carrier status and grade 3/4 neutropenia (Figure 3). However, substantial heterogeneity was observed (I^2^ = 75%, Chi^2^ = 12.21, p = 0.007), rendering the overall pooled estimate uninterpretable.

The test for subgroup differences was statistically significant (Chi^2^ = 7.82, df = 2, p = 0.02, I^2^ = 74.4%), confirming that the direction and magnitude of the genotype–neutropenia association differed across ancestral populations.

Two studies contributed to the European/Caucasian subgroup: Iwata non-Asian (n = 530, predominantly white) and Peruzzi (n = 195, self-reported Caucasian). T-carrier status showed a trend toward increased risk of grade 3/4 neutropenia, with a pooled OR of 1.87 (95% CI 0.87–4.00, p = 0.11). The two individual study estimates were directionally concordant (Iwata: OR 1.39, 95% CI 0.90–2.16; Peruzzi: OR 3.11, 95% CI 1.22–7.91), though moderate heterogeneity was present (I^2^ = 57%, p = 0.13). Peruzzi reached statistical significance individually, while Iwata did not. The neutropenia event rate among T-carriers was 25.1% (88/350) in Iwata and 23.4% (30/128) in Peruzzi, compared with 19.4% (35/180) and 9.0% (6/67) in CC homozygotes, respectively.

Only the Iwata Asian subgroup (n = 122) contributed to the East Asian subgroup. The OR was 0.57 (95% CI 0.18–1.78, p = 0.33), suggesting a non-significant trend in the opposite direction—CC homozygotes had numerically higher neutropenia rates (66.7%, 10/15) than T-carriers (53.3%, 57/107). The small number of CC homozygotes (n = 15) limited statistical power.

Ibrahim Mohamed Ebid (n = 95, Egyptian) was the sole contributor to the North African/Middle Eastern subgroup. The OR was 0.33 (95% CI 0.12–0.92, p = 0.03), indicating that T-carrier status was significantly associated with lower risk of grade 3/4 neutropenia. The neutropenia rate was 67.6% (25/37) in T-carriers versus 86.2% (50/58) in CC homozygotes. The direction of effect was opposite to that observed in the European/Caucasian subgroup. Of note, the genotype distribution in this study deviated from Hardy-Weinberg equilibrium (p = 0.0007), and the outcome was cumulative neutropenia rather than day-14 neutropenia.

### 3.4 Association between *ERCC1*_rs11615 and grade 3/4 neutropenia

**FIGURE 4:**
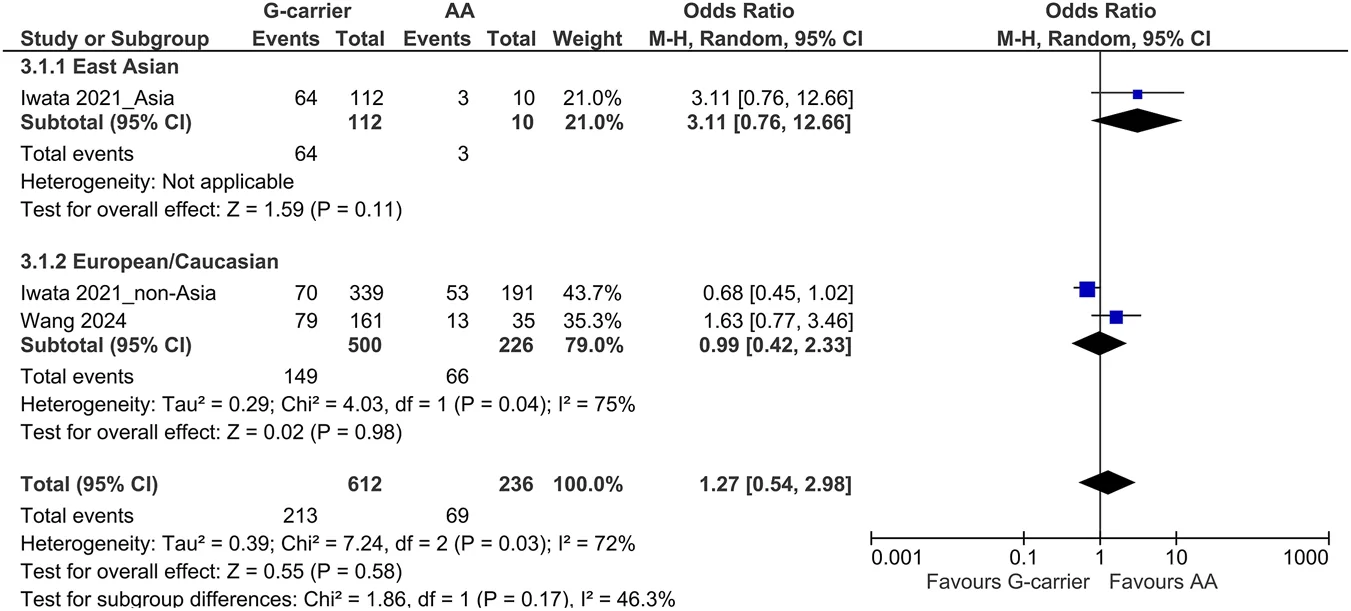
Forest plot of the association between *ERCC1* rs11615 G-carrier status (GG + AG vs. AA) and grade 3/4 neutropenia in CDK4/6 inhibitor–treated breast cancer patients, stratified by ancestry subgroup (Mantel–Haenszel random-effects model).

Two studies (three population entries) reported on the association between *ERCC1* rs11615 and grade 3/4 neutropenia: Iwata 2021, contributing an East Asian (n = 122) and a European/Caucasian (non-Asian, n = 530) subgroup from the PALOMA-2/3 palbociclib cohort; and Wang 2024 (n = 196, US, palbociclib), classified as ancestry unspecified (US cohort; racial composition not reported by the original study; genotype frequencies were intermediate between European and East Asian reference values). A sensitivity analysis excluding the Wang cohort was performed to assess the robustness of the *ERCC1* rs11615 findings. Wang’s 2 × 2 table was back-calculated from reported codominant ORs and known genotype group totals (G-carrier = 161, AA = 35). Data were analyzed using a dominant model for the G allele (G-carrier [GG + AG] versus AA) with Mantel-Haenszel random-effects weighting. The overall pooled OR across all three entries (612 G-carriers, 236 AA homozygotes; 282 total events) was 1.27 (95% CI 0.54–2.98, p = 0.58), with substantial heterogeneity (I^2^ = 72%, Chi^2^ = 7.24, p = 0.03) (Figure 4). The test for subgroup differences was not statistically significant (Chi^2^ = 1.86, df = 1, p = 0.17, I^2^ = 46.3%).

Iwata Asian was the sole contributor for the Asian subgroup (64/112 G-carriers versus 3/10 AA; OR 3.11, 95% CI 0.76–12.66, p = 0.11, weight 21.0%). The wide confidence interval reflects the very small number of AA patients (n = 10), consistent with the low A-allele frequency in East Asian populations. The neutropenia rate among G-carriers was 57.1% (64/112) versus 30.0% (3/10) among AA homozygotes.

Two studies contributed to the European/Caucasian subgroup: Iwata non-Asian (70/339 G-carriers versus 53/191 AA; OR 0.68, 95% CI 0.45–1.02, weight 43.7%) and Wang (79/161 versus 13/35; OR 1.63, 95% CI 0.77–3.46, weight 35.3%). The two estimates pointed in opposite directions: Iwata suggested a borderline protective effect of G-carrier status (OR 0.68, p = 0.06), whereas Wang suggested a non-significant risk effect (OR 1.63, p = 0.20). The pooled European/Caucasian subgroup OR was 0.99 (95% CI 0.42–2.33, p = 0.98), with substantial heterogeneity (I^2^ = 75%, Tau^2^ = 0.29, Chi^2^ = 4.03, p = 0.04). The essentially null pooled estimate (OR 0.99) indicates that the borderline signal observed in Iwata’s non-Asian subgroup alone was not replicated by Wang and does not withstand pooling.

### 3.5 Association between *ERCC1*_rs3212986 and grade 3/4 neutropenia

**FIGURE 5:**
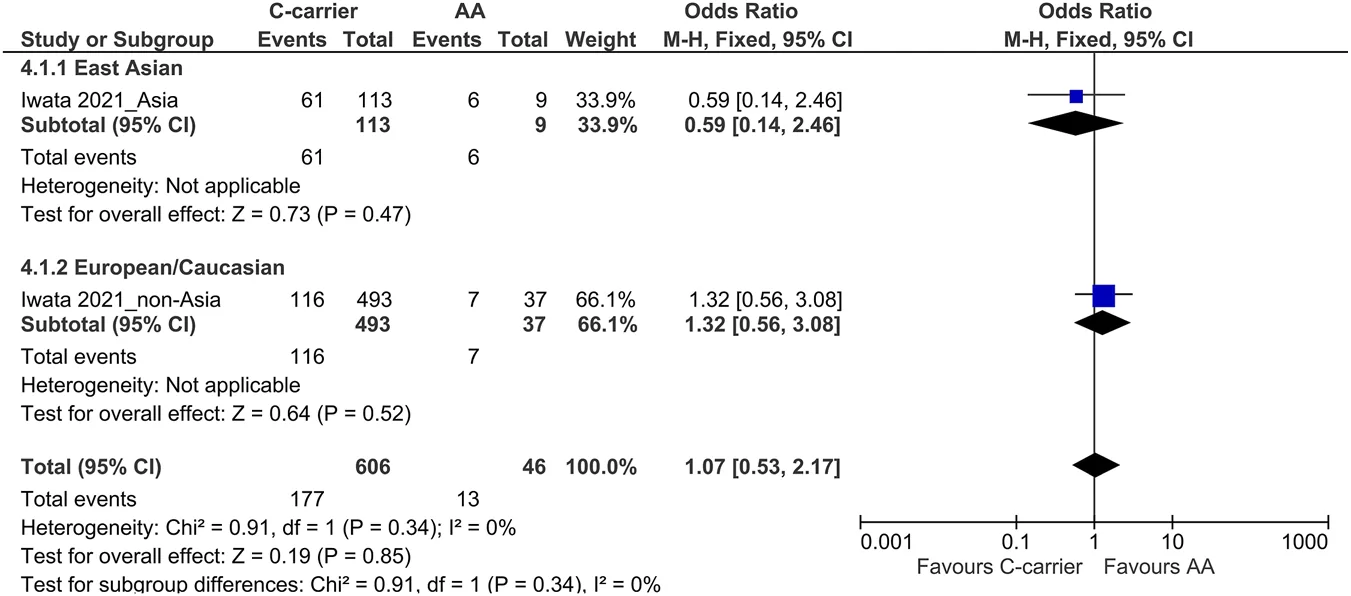
Forest plot of the association between *ERCC1* rs3212986 C-carrier status (CC + AC vs. AA) and grade 3/4 neutropenia in CDK4/6 inhibitor–treated breast cancer patients, stratified by ancestry subgroup (Mantel–Haenszel fixed-effect model).

As with rs11615, only Iwata 2021 reported genotype-stratified data for *ERCC1* rs3212986, contributing two ancestry subgroups. Data were analyzed using a dominant model (C-allele carrier [CC + AC] versus AA homozygotes) with Mantel-Haenszel fixed-effect weighting. The overall pooled OR was 1.07 (95% CI 0.53–2.17, p = 0.85), with no heterogeneity (I^2^ = 0%, Chi^2^ = 0.91, p = 0.34) (Figure 5). No statistically significant association between rs3212986 genotype and grade 3/4 neutropenia was observed. Among Asian patients (61/113 C-carriers versus 6/9 AA; OR 0.59, 95% CI 0.14–2.46, p = 0.47), no significant association was found. The AA group was very small (n = 9), limiting statistical power. In non-Asian patients (116/493 versus 7/37; OR 1.32, 95% CI 0.56–3.08, p = 0.52), the OR was slightly above unity but far from significance. This is consistent with Iwata’s original univariate analysis, which reported no significant association for any rs3212986 genotype comparison in either population (all p > 0.70).

## 4 Discussion

This systematic review and meta-analysis is the first to pool genotype-level data from independent studies to evaluate the association between germline pharmacogenomic polymorphisms and CDK4/6i–induced neutropenia. Four SNPs in two genes were analyzed across four studies encompassing 1,138 patients from Europe, East Asia, and North Africa. While no SNP demonstrated a significant overall association when pooled across all populations, this null result may reflect ancestry-dependent heterogeneity in the genotype–neutropenia relationship. In the European/Caucasian subgroup, *ABCB1* rs1045642, T-allele carrier status showed a statistically significant association with increased grade 3/4 neutropenia risk with a concordant trend for *ABCB1* rs1128503. Both associations appeared to reverse direction in East Asian and North African/Middle Eastern populations, although these subgroups were each represented by a single study and the observed differences may reflect study-specific characteristics rather than true biological variation.

The parallel findings for rs1128503 and rs1045642 are directionally consistent with an effect of the *ABCB1* locus on CDK4/6i–induced neutropenia. It is important to emphasize, however, that rs1128503 and rs1045642 are in strong linkage disequilibrium (Hoffmeyer et al., 2000; Horinouchi et al., 2002); their concordant results therefore do not constitute two statistically independent replications but rather reflect two correlated markers tagging the same underlying signal, and the effective amount of independent evidence is correspondingly smaller than the number of SNPs analyzed. Together with rs2032582, these variants form the well-characterized *ABCB1* 1,236T–2677T(A)–3435T haplotype associated with reduced P-glycoprotein (P-gp) expression and function (Salama et al., 2006; Vivona et al., 2014). Peruzzi et al. confirmed this linkage in their cohort and demonstrated that the haplotype analysis produced the strongest associations: homozygous carriers of the variant haplotype had significantly higher risk of day-14 neutropenia and early dose-limiting toxicities, with a trend toward higher ribociclib trough concentrations (934.0 ng/mL versus 668.0 ng/mL in non-carriers) (Peruzzi et al., 2023). This suggests that the individual SNP associations observed in the present meta-analysis are components of a larger haplotype effect that may be more clinically meaningful than any single variant. Importantly, the concordant direction of effects for rs1128503 and rs1045642 likely reflects a shared underlying haplotype effect rather than two independent associations. A key limitation of the present meta-analysis is that haplotype-level pooling was not feasible because only Peruzzi et al. reported haplotype-stratified outcome data; the other included studies reported only individual SNP-level results. Future prospective studies should incorporate *ABCB1* haplotype analysis (including rs2032582) rather than single-SNP analyses, as the haplotype effect may be more clinically informative and actionable than individual variant associations.

The biological mechanism linking *ABCB1* variants to neutropenia is coherent. P-gp is an ATP-dependent efflux transporter expressed at the intestinal epithelium, hepatocyte canalicular membrane, and renal tubular cells (Hodges et al., 2011). All three CDK4/6i are established P-gp substrates in preclinical models (de Gooijer et al., 2015; Martínez-Chávez et al., 2019; Martínez-Chávez et al., 2022; Raub et al., 2015). The rs1128503 and rs1045642 variants, although synonymous, have been associated with altered mRNA stability and reduced P-gp function—particularly as part of the T-T-T(A) haplotype (Salama et al., 2006; Vivona et al., 2014; Keskitalo et al., 2008). Reduced intestinal P-gp efflux would increase oral bioavailability of CDK4/6i, leading to higher systemic exposure and greater myelosuppression. This mechanistic chain is supported at the individual patient level by Roncato et al., who described a palbociclib-treated patient carrying the homozygous low-function *ABCB1* haplotype who exhibited both drug overexposure and recurrent grade 4 neutropenia (Roncato et al., 2022), and by Maeda et al., who found that *ABCB1* rs2032582 homozygous variant carriers had higher abemaciclib concentrations and greater treatment intolerance (Maeda et al., 2022).

A notable discordance exists, however, between the genotype–neutropenia and genotype–exposure relationships. Iwata et al. reported no association between *ABCB1* genotype and palbociclib trough concentrations (Iwata et al., 2021), and Peruzzi et al. found no significant palbociclib differences across haplotype groups, despite the trend for ribociclib (Peruzzi et al., 2023). This raises the possibility that P-gp variants affect local tissue-level drug concentrations - particularly in the bone marrow microenvironment - without measurably altering systemic trough levels. Alternatively, the association may be mediated through linkage disequilibrium with uncharacterized functional variants.

The ancestry-dependent reversal of effect direction is an observation that warrants further investigation, although it must be interpreted with caution given that each ancestry subgroup was represented by a single study population and the observed differences may reflect study-specific characteristics rather than true ancestry-related biology. For both *ABCB1* SNPs, T-carrier status was associated with increased neutropenia risk in Europeans but significantly decreased risk in East Asians (rs1045642: OR 0.31, p = 0.009) and North African/Middle Eastern patients (rs1128503: OR 0.33, p = 0.03). Several factors may explain this heterogeneity. First, allele frequencies differ substantially: the rs1128503 T-allele frequency is approximately 65% in East Asians versus 43% in Europeans, while rs1045642 shows the inverse pattern (Iwata et al., 2021). The reference genotype (CC) therefore represents a common group in one population and a rare group in another, fundamentally altering the statistical properties of the comparison. Second, Asian patients have higher baseline neutropenia rates regardless of genotype (54.9% versus 23.2% in Iwata (Iwata et al., 2021)), driven by lower baseline ANC and body weight, which may overwhelm modest pharmacogenetic effects. Third, population-specific linkage disequilibrium patterns mean the same SNP may tag different functional haplotypes across ancestral backgrounds (Wolking et al., 2015). Lai et al. confirmed through the Taiwan Biobank (n = 108,801) and other population databases that these allele frequency differences are genuine population-level phenomena (Lai et al., 2024). The reversed direction in the Egyptian cohort (Ibrahim Mohamed Ebid et al., 2025) must be interpreted with additional caution due to significant Hardy-Weinberg equilibrium deviation (p = 0.0007), a different outcome timepoint (cumulative versus day-14 neutropenia), and the distinct North African genetic architecture.

The minor allele frequencies (MAF) of the analyzed SNPs vary substantially across ancestral populations, a factor that directly affects the statistical power and interpretation of ancestry-stratified analyses. For *ABCB1* rs1128503 (c.1236C>T), the T-allele frequency is approximately 43% in European populations, 65% in East Asian populations, and 17% in African populations according to the 1000 Genomes Project (1000 Genomes Project Consortium et al., 2015) and the Genome Aggregation Database (gnomAD) (Karczewski et al., 2020), consistent with earlier pharmacogene reports (Hodges et al., 2011; Wolking et al., 2015). Consequently, the CC reference genotype used in the dominant model represents a common group in Europeans (∼32% of individuals) but a relatively rare group in East Asians (∼12%), which reduces the precision of subgroup estimates in the latter population. Conversely, for *ABCB1* rs1045642 (c.3435C>T), the T-allele frequency is higher in Europeans (∼52%) than in East Asians (∼40%) and considerably lower in African populations (∼19%) (Hoffmeyer et al., 2000; Chowbay et al., 2003; 1000 Genomes Project Consortium et al., 2015; Karczewski et al., 2020), meaning the CC reference genotype is less frequent in Europeans (∼23%) than in East Asians (∼36%). This inverse MAF pattern between the two *ABCB1* SNPs across populations is consistent with the well-documented linkage disequilibrium structure of the *ABCB1* locus (Horinouchi et al., 2002; Wolking et al., 2015) and may partly explain why subgroup-specific effect estimates differ in magnitude despite concordant effect directions within each ancestry group. The genotype distributions observed in the included studies were broadly consistent with these reference values: in Iwata’s cohort, the rs1045642 CC genotype frequency was 13.2% in Asians versus 23.0% in non-Asians, while the rs1128503 CC frequency was 6.6% in Asians versus 34.0% in non-Asians (Iwata et al., 2021). In Ibrahim Mohamed Ebid Egyptian cohort, the rs1128503 CC genotype was predominant (61.1%), consistent with the low T-allele frequency reported in North African/Middle Eastern populations (Ibrahim Mohamed Ebid et al., 2025). For *ERCC1* rs11615, the G-allele (corresponding to C on the coding strand) frequency is approximately 37% in Europeans and 27% in East Asians (1000 Genomes Project Consortium et al., 2015; Karczewski et al., 2020), yielding AA homozygote reference groups of roughly 40% and 53%, respectively. For *ERCC1* rs3212986 (c.8092C>A), the A-allele frequency is relatively similar across populations (∼27% in Europeans and ∼30% in East Asians) (1000 Genomes Project Consortium et al., 2015; Karczewski et al., 2020), resulting in more balanced reference group sizes and contributing to the low heterogeneity observed for this SNP. These inter-ethnic MAF differences have important implications: when the reference genotype group is small, odds ratio estimates become unstable, confidence intervals widen, and the risk of spurious associations increases, which underscores the need for adequately powered, ancestry-stratified replication studies.

The borderline *ERCC1* rs11615 signal observed in Iwata 2021 alone was not replicated when Wang 2024 was added: the pooled European/Caucasian estimate became essentially null, and the subgroup difference between ancestry groups lost significance. This non-replication is consistent with the “winner’s curse” phenomenon common in candidate gene pharmacogenomics (Lai et al., 2024) and with the weaker biological rationale for *ERCC1*: whereas *ABCB1* has a direct pharmacokinetic link to CDK4/6 inhibitor exposure, *ERCC1* encodes a nucleotide excision repair protein whose relevance to cell cycle arrest–mediated neutropenia (as opposed to DNA damage–mediated myelosuppression) is mechanistically indirect (Hu et al., 2016; Tsuji et al., 2016). *ERCC1* rs3212986 showed no association in any subgroup.

The four SNPs now form a clear mechanistic hierarchy: *ABCB1* variants (pharmacokinetically proximal, replicating across independent studies) > *ERCC1* rs11615 (mechanistically indirect, non-replicating, null after pooling) = *ERCC1* rs3212986 (null). This gradient is biologically intuitive and suggests that *ABCB1* is currently the most promising candidate locus for further investigation in the CDK4/6 inhibitor pharmacogenomic context, although this preliminary conclusion is based on a limited evidence base and requires confirmation in larger, adequately powered studies. Notably, Wang (38) identified borderline associations for two SNPs not examined in the present meta-analysis: *ABCG2* rs2231137 (OR 4.14, p = 0.052) and *SULT2A1* rs182420 (OR 4.33, p = 0.042), suggesting that pharmacogenomic determinants of CDK4/6 inhibitor toxicity extend beyond the *ABCB1* and *ERCC1* loci and that alternative metabolic pathways warrant investigation.

Several limitations should be acknowledged. The principal limitation of this synthesis is the small number of eligible studies, which reflects the emerging nature of CDK4/6 inhibitor pharmacogenomics rather than a restrictive search: a comprehensive search of three databases without language or date restrictions, conducted under a registered protocol and in accordance with PRISMA and HuGENet guidance, identified only four eligible studies, because genotype-stratified neutropenia data for these agents have begun to appear only recently. This scarcity has direct statistical consequences that warrant cautious interpretation. Confidence intervals are wide, between-study heterogeneity estimates (I^2^) are themselves imprecise when derived from few studies, and formal assessment of publication bias was precluded because no comparison included the minimum of ten studies recommended for funnel-plot or Egger’s testing. Several ancestry subgroups were represented by a single study population, so their estimates cannot distinguish genuine ancestry-related effects from study-level confounders such as differing dose-intensity protocols, concomitant medications, baseline absolute neutrophil count distributions, or outcome timepoints. For these reasons, the pooled estimates should be regarded as preliminary. To extract the maximum reliable signal from the available data, we analysed each SNP separately with pre-specified ancestry stratification rather than reporting a single pooled estimate, performed a sensitivity analysis excluding the cohort with unverified ancestry, appraised study quality with a genetics-specific instrument (Q-GENIE), and reported the reconstruction of 2 × 2 tables transparently. Outcome definitions were not fully harmonized, with Ibrahim Mohamed Ebid assessing cumulative neutropenia rather than the day-14 timepoint used by Iwata and Peruzzi (Peruzzi et al., 2023; Iwata et al., 2021; Ibrahim Mohamed Ebid et al., 2025). One study pooled all three CDK4/6i (Peruzzi et al., 2023), though drug-independent effects were confirmed in multivariate analysis. The 2 × 2 tables for Peruzzi were back-calculated from reported ORs. The Hardy-Weinberg deviation in (Ibrahim Mohamed Ebid et al., 2025) raises genotyping quality concerns. Publication bias could not be formally assessed given the small number of studies. The racial composition of the Wang cohort (Wang et al., 2024) was not reported; this cohort has therefore been reclassified as ancestry unspecified, and a sensitivity analysis excluding it was performed to assess the robustness of the *ERCC1* rs11615 results. Furthermore, each ancestry subgroup in the stratified analyses was represented by a single study population, and the observed subgroup differences may therefore reflect study-specific characteristics rather than true ancestry-related biological effects. No published pharmacogenomic data for abemaciclib-treated patients met our inclusion criteria, limiting generalizability to palbociclib and ribociclib. The inability to perform haplotype-level meta-analysis, owing to the absence of haplotype-stratified outcome data in all studies except Peruzzi et al., is a further limitation. The reconstruction of 2 × 2 genotype–event tables from published summary statistics introduces an additional source of uncertainty and future primary studies should be encouraged to report complete 2 × 2 genotype–event tables for all genetic models analyzed, which would eliminate the need for *post hoc* reconstruction and improve the reliability of subsequent meta-analytic pooling.

In conclusion, this meta-analysis provides preliminary evidence suggesting that *ABCB1* rs1045642 T-allele carrier status may be associated with CDK4/6i–induced grade 3/4 neutropenia in European/Caucasian patients, with a concordant trend for rs1128503 and biological plausibility through the P-glycoprotein efflux mechanism. These findings should be interpreted as hypothesis-generating. The limited and recent evidence base means that the present synthesis is best viewed as an initial quantitative baseline for a developing field, to be updated as further genotype-stratified cohorts are reported. Prospective validation in adequately powered, ancestry-stratified cohorts—incorporating *ABCB1* haplotype analysis, concurrent pharmacokinetic sampling, and standardized day-14 neutropenia assessment—is warranted to test the specific hypotheses generated here, in particular the association of *ABCB1* rs1045642 in European/Caucasian patients and its apparent ancestry-dependent reversal. The observed associations appear to be ancestry-dependent, although the ancestry subgroups were each represented by single study populations, and the observed heterogeneity may reflect study-specific characteristics rather than true biological differences. The *ERCC1* signal, previously borderline in a single study, was not confirmed upon pooling with an independent cohort. Prospective validation in adequately powered, ancestry-stratified cohorts - incorporating *ABCB1* haplotype analysis, concurrent pharmacokinetic sampling, and standardized day-14 neutropenia assessment - is warranted to determine whether pharmacogenetic testing can contribute to individualized CDK4/6i dosing and toxicity management.

## Data Availability

The original contributions presented in the study are included in the article/Supplementary Material, further inquiries can be directed to the corresponding author.
